# Effect of Cutting Conditions on Roughness and Cutting Force When Machining a Freeform Surface with Barrel Tools

**DOI:** 10.3390/ma19050988

**Published:** 2026-03-04

**Authors:** Martin Reznicek, Cyril Horava, Jakub Zajicek, Martin Ovsik

**Affiliations:** Faculty of Technology, Tomas Bata University in Zlin, Vavreckova 5669, 760 01 Zlin, Czech Republic; mreznicek@utb.cz (M.R.); c_horava@utb.cz (C.H.); j_zajicek@utb.cz (J.Z.)

**Keywords:** barrel tools, roughness, cutting force, freeform surface

## Abstract

Barrel tools are relatively new tools that use atypical geometries to achieve shorter production times and improve surface quality. They have been increasingly used in the finishing operations industry, where they are gaining more and more popularity. For their optimal use, it is necessary to know how these tools behave during work in terms of how they load the machined product and what surface qualities they can achieve. For this reason, this study was conducted to compare two tools when machining a free surface. The obtained surface quality and the force load caused by the tool were evaluated. It was found that barrel tool machining results in a heterogeneous surface caused by different cutting speeds along the length of the tool and that the two obtained regions show differences in the obtained roughness. Even though the operation was classified as a finishing process, a difference of up to 30% was identified in the cutting forces acting on the tool and the workpiece.

## 1. Introduction

Freeform or shaped surfaces are complex surfaces with different shapes/dimensions along three principal axes and have no translational symmetry and axes of rotation [1]. With the development of geometric modeling techniques, these complex surfaces can be represented and proposed for industrial production [2]. They are widely used in the automotive, aerospace, and plastics industries [1,3].

The specified surface quality is key to these parts’ wear resistance, ductility, and fatigue strength. Therefore, controlling surface roughness is a key factor in machining [4]. Part quality is characterized by two aspects: dimensional accuracy and surface roughness. While the former relates to macro-geometric errors and component interchangeability, surface roughness is related to micro-geometric errors. It has essential implications for component life and interoperability with other components. During the milling process, the tool removes material in the form of chips and leaves traces on the surface of the workpiece. These subtle bumps, peaks, and troughs create a roughness pattern on the workpiece that is specific to the type of operation (turning, milling, and grinding operations, among others) and related to the cutting parameters. Key mechanical properties required for components include contact stiffness, wear and corrosion resistance, and fatigue strength [3,4].

Milling freeform surfaces using CNC machines is a technically demanding and expensive operation that requires careful selection of the cutter type. This choice significantly influences the machined surface’s quality and the operation’s overall duration. Machining these surfaces requires a specific procedure as the tool and workpiece meet in uneven planes, distinguishing these surfaces from those in the standard flat milling process and placing high demands on the accuracy and quality of the resulting surface. For these purposes, ball mills are often used to ensure smooth contact between the tool and the workpiece. This constant interaction is essential for optimal performance and machining accuracy, especially for freeform surfaces, which are common in advanced design applications [5,6,7,8]. The use of ball mills in this area was investigated by Grešová et al. [9]. They compared the use of three-axis and five-axis machining operations in producing free surfaces. They concluded that five-axis operations require longer production times, but the resulting surface quality is better. However, this does not mean that ball mills are the most suitable.

The working edge of the ball mill is located in the ball end area, which can adapt well to the contour of the parts, resulting in high machining accuracy [3,10]. Another advantage is the simple planning of the tool path and the easy handling of cutter collisions. However, the ball end mill is in point contact with the surface; this creates a serrated finish, leaving little tip height between two consecutive passes. The number of tool paths is vast for large surfaces with slight curvature [10,11]. The process efficiency is low, especially for freeform surface machining [12]. This leads to long machining times and high costs that are not affordable for small businesses [4]. For these reasons, barrel instruments are being increasingly used. In their article, Luo [12] described the advantages of barrel cutters compared to ball cutters. Not only does their use improve the surface roughness, but it also significantly shortens the machining time due to a larger contact area with the workpiece.

Barrel milling is a machining process with curved contact, the error of which is more difficult to control due to the large area of the tool involved in the cutting and the complex position between the tool and the machined surface [3]. The advantages of this curved contact were described by Wang et al. [3], who managed to further increase the efficiency with their global space-based wide strip calculation method. The barrel milling process can achieve higher machining efficiency and better surface quality than a ball-nose cutter [3,11]. On the other hand, in side milling with a large barrel radius, the cutting area is expanded even at a small depth of cut compared to finishing with a ball-nose milling cutter. The large cutting forces in this process can cause tool deflection, which would deteriorate the machining accuracy. Therefore, the cutting parameters and the inclination of the milling cutter axis relative to the machined surface should be evaluated regarding the cutting forces [13]. Burek [14] and his team simulated cutting forces generated during machining with barrel cutters and also emphasized the importance of studying cutting forces. They revealed significant variations in cutting troops depending on the tool geometry. The selection of a suitable tool for geometries was addressed by Fan-Jun et al. [15], who compared the suitability of individual tool geometries for machining free surfaces.

None of the studies investigated the influence of cutting conditions on the magnitude of cutting forces and the obtained surface roughness. The research has shown that these areas are key for the industry, as barrel tools are increasingly used in finishing operations. For this reason, this work was created to investigate this influence.

Existing studies have primarily focused on issues related to form accuracy. In the evaluation of surface quality, the inherent heterogeneity of the surface has not been explicitly considered, and the investigated surface has been evaluated as a single uniform entity. The present study addresses surface heterogeneity through localized data acquisition and evaluation at selected surface regions exhibiting different parameter values, followed by a comparative analysis.

To ensure objective measurement and reliable data evaluation, an experimental methodology was developed based on a comprehensive assessment of the cutting tools, the sample material, and the milling process. The acquired data were subsequently subjected to statistical analysis, followed by interpretation of the results.

## 2. Materials and Methods

A block of material 1.1730 was chosen for the experiment. It is an unalloyed tool steel with excellent machinability and good core toughness. Due to its properties, it does not require heat treatment. It is used for producing unhardened mold parts (plates and frames) and die sets. Its composition is given in Table 1.

### 2.1. Cutting Tools

**Figure 1:**
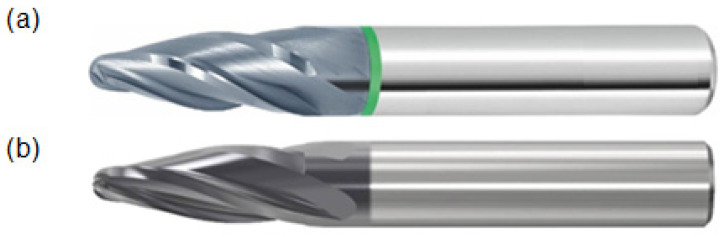
Barrel tools: (**a**) JH734100X2R2R85.0Z4 SIRA from SecoTools; (**b**) 207525 10/90 from Hoffman Group.

The experiment aimed to compare two-barrel tools, one with the trade mark JH734100X2R2R85.0Z4 SIRA from SecoTools (Brno, Czech Republic), shown in Figure 1b, and the other with the trade mark 207525 10/90 from Hoffman Group (Nürnberg, Germany), shown in Figure 1a. Both are tangential tools that are suitable for machining hard-to-reach cavities, e.g., the production of mold cavities.

Although both tools are of the same type, they have minor differences due to customer requirements or company experience. The most important properties of the individual tools and their differences are listed in Table 2. As mentioned above, the tools are intended for the same machining operations. This article will describe the differences the tools achieve because even such minor differences can lead to significant changes that can decide which tool will be purchased.

For easier orientation, the tools will be marked with their effective radius. The JH734100X2R2R85.0Z4 SIRA tool will be labeled R85, and the 207,525 10/90 tool will be labeled R90.

The experiment was designed to describe real machining situations. Therefore, the cutting conditions of the experiment were chosen according to the recommendations of the tool manufacturers. Table 3 contains these selected conditions. The recommended cutting parameters for the investigated material are specified by the tool manufacturer. The cutting conditions presented in Table 3 were established to cover the entire spectrum of these recommendations while taking into account the number of planned experimental runs.

Since these conditions will most often be observed in practice, knowing their influence on the machining process is desirable.

### 2.2. Machining Tool

**Figure 2:**
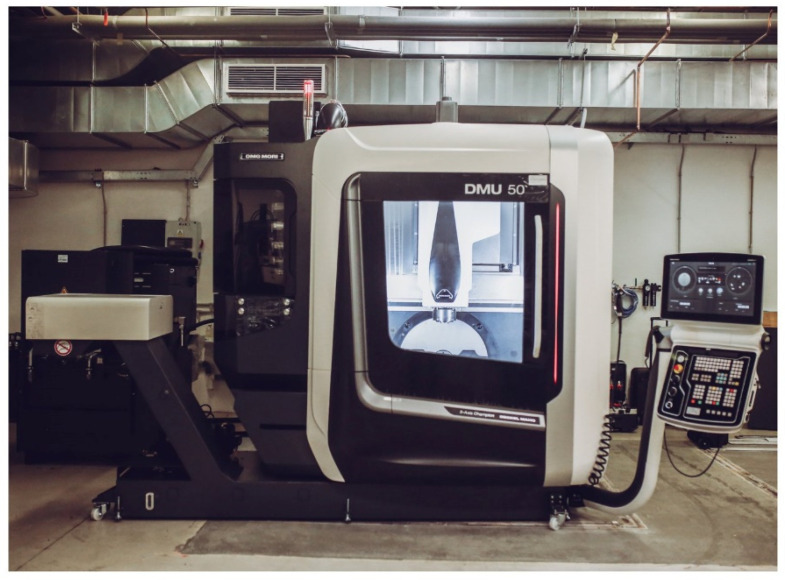
DMU 50.

The experiment was performed on a CNC machine, DMU 50 (Figure 2), from DMG MORI (Brno, Czech Republic), which has a five-axis milling center. The spindle enables displacements in the X, Y and Z axes. The clamping table, in turn, enables rotation in the B and C axes. The maximum spindle speed is 15,000 rpm, and the maximum feed speed is 30 m/min.

### 2.3. Measuring Equipment

Several measuring devices were used to meet the needs of the experiment. The cutting forces were measured using a dynamometer 9129AA from the Kistler company (Sindelfingen, Germany), whose clamping surface has dimensions of 90 × 105 mm. A NewView 800 3D profilometer from Zygo (Middlefield, CT, USA) was used to evaluate the obtained roughness.

### 2.4. Cutting Geometry

**Figure 3:**
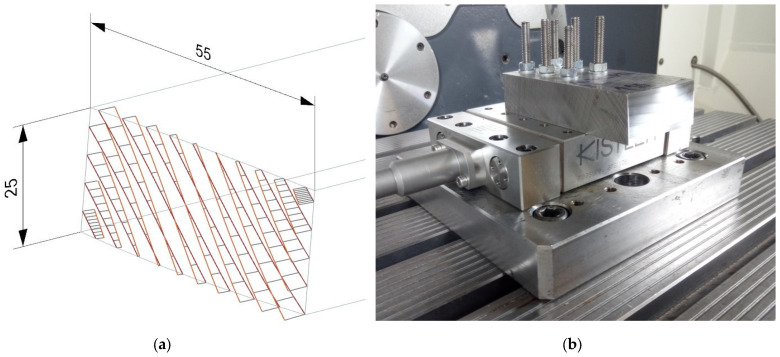
(**a**) Freeform surface; (**b**) clamped test body.

A free surface (Figure 3a) with dimensions of 55 × 25 mm was designed for the experiment. The surface was created on a test body with a total length of 110 mm. An analysis of the surface curvature was performed on the image of the test geometry to better describe the shape of the created free surface.

Using holes made in the body, it was clamped to the dynamometer using threaded rods, as shown in Figure 3b. The test body was placed on these rods and clamped with nuts. This clamp was chosen because it provided the best contact surface with the dynamometer. It also created a tight fit to ensure that the test body does not vibrate, which could lead to skewed results, which is possible using different preparations. In addition, these would increase the assembly’s weight, increasing centrifugal forces during turning and tilting during machining. Another advantage of this method is the easy and quick exchange of test bodies. The test body was thus clamped to the dynamometer, which was fastened to the work table of the machine tool via the clamping plate. After the machining operation, roughness was always evaluated and the program from Siemens (Munich, Germany) was used to generate tool paths. This software offers a swarf drive function intended for generating barrel tool paths. The inclination of the tools from the Z axis was set to 10°. In simulations, this value proved to be the most suitable for achieving optimal results in terms of roughness. For both operations, the maximum scallop height was set to 0.1 mm in the setting conditions.

It can be seen from the compression that there are practically no differences between the tracks generated for the tools. Both tools required eight paths to machine the whole surface. The toolpaths were generated so that only climb milling was used. This was chosen because it leads to better surface quality; since machining with barrel tools is a finishing operation, achieving the best possible surface is desirable. In addition, the intermittent cutting process can be better analyzed.

**Figure 4:**
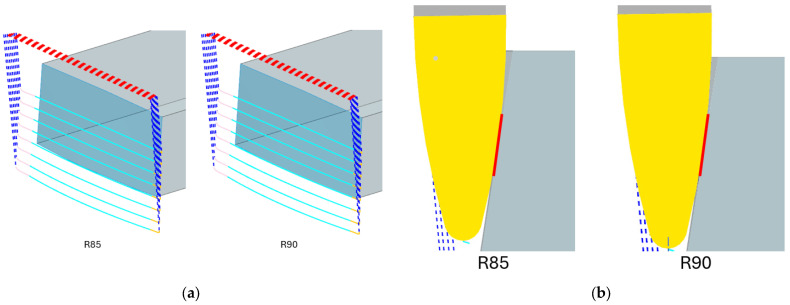
(**a**) Tools paths; (**b**) contact area.

Figure 4b shows how much contact was achieved between the tool and the workpiece (highlighted by the red line). The 4th track in the middle of the workpiece is shown in both cases. From this view and the total number of tracks shown above, it is clear that barrel tools can significantly increase production efficiency due to the size of the contact area. However, the contact surface could change during machining, as the program could evaluate that it is necessary to tilt the tool to maintain the set accuracy or to prevent a collision.

## 3. Results

As mentioned above, the focus of this work was monitoring the force loading on the workpiece during machining and evaluating the dependence of the cutting conditions on the resulting surface roughness. A total of ten repetitions were performed for each measurement to verify the repeatability of the experimental procedure.

### 3.1. Cutting Forces

**Figure 5:**
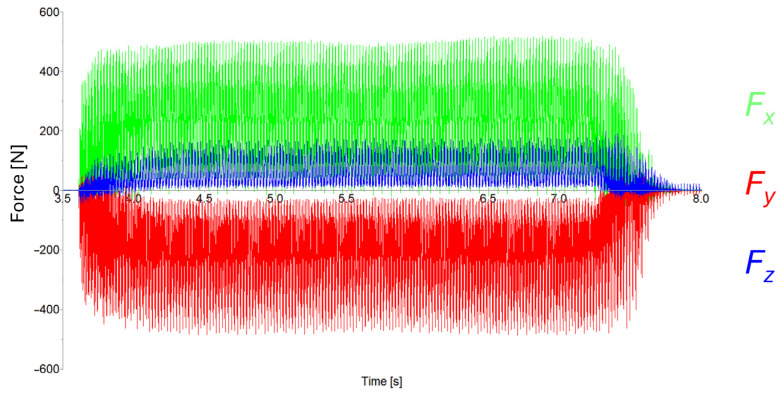
Cutting forces.

Cutting forces were evaluated using DynoWare 3.2.5.0 software from Kisstler (Winterthur, Switzerland). In Figure 5, it is possible to see how the software represents the force load acting on the workpiece. The positive or negative sign of the recorded values results from the orientation of the measurement system’s coordinate axes relative to the direction of the mechanical load. This is due to the orientation system of the dynamometer, which did not correspond with the orientation system of the machine.

Cutting forces act on the workpiece in three basic axes (X, Y and Z). Considering the use of five-axis machining and the resulting tilting during the machining process, the evaluation of cutting forces in individual axes would be quite problematic and therefore, for this experiment, the total cutting force was evaluated, which can be obtained from the following equation:

(1) $$\vec{F_{C}}=\sqrt{\vec{{F_{X}}^{2}}+\vec{{F_{Y}}^{2}}+\vec{{F_{Z}}^{2}}}$$

**Figure 6:**
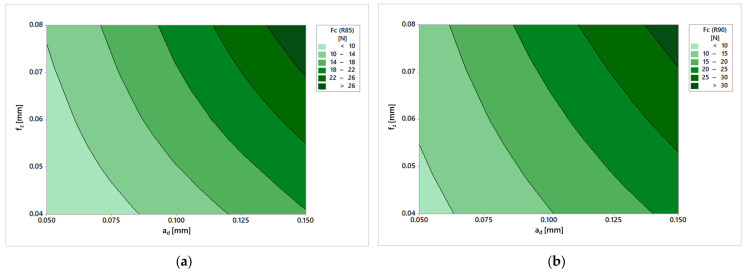
Cutting forces: (**a**) R85; (**b**) R90.

The total cutting forces were then plotted on the graph (shown in Figure 6a). It can be seen from the figure that the cutting forces are dependent not only on the machining allowance, but also on the feed per tooth. The increase in total cutting force is most noticeable when changing the machining allowance. On the contrary, when the feed changes, it is less significant.

Similar trends were observed for the second tool (Figure 6b). This tool generally achieved higher values of cutting forces, albeit only slightly.

Since both tools were new and had never been used before the experiment, it is possible to attribute this slight difference to the differences in geometry, which stems from different values of the radius of the tool profile and the helix angle. The impact of the tool itself probably also had an effect. As mentioned above, different manufacturers have different experiences, which can translate into differences in tool sharpening. To confirm this assumption, separate studies would be necessary, but lower values of cutting forces are generally associated with a lower value of the cutting-edge angle. The R85 tool may not load the workpiece with such large cutting forces, but it may have a shorter service life. The differences in forces are more apparent and are shown in Table 4, where the percentage differences between the obtained values are also shown.

Figure 6 shows the dendrogram representation of the cutting force (Fc) for the R85 and R90 tool geometries. The plotted force bands corresponding to the individual cutting conditions defined by the depth of cut (ad) and feed per tooth (fz) are clearly distinguishable, allowing for straightforward interpretation for machining practice. The recorded values demonstrate strong internal consistency.

The results obtained from the statistical evaluation are listed in the following tables. After identification and exclusion of outliers, the arithmetic mean of the measured values was calculated. Continuous data validation was carried out during the experimental phase to prevent systematic errors.

It can be seen from the table that in all cases, the R90 tool achieved higher values. However, the percentage trend is interesting: with increasing allowance for ma-chinning, the difference decreases and, at the same time, decreases with increasing feed per tooth. The difference between the lowest feed and the smallest addition is over 30 percent, while at the highest values of the cutting conditions, it only reached 11 percent. Another important finding is that the total cutting forces obtained in this experiment were relatively small values. When processing subtle products with a small thickness, damage may occur due to cutting forces. However, when the manufacturer’s recommended cutting conditions were used, they caused minimal loading during finishing operations with barrel tools.

### 3.2. Surface Quality

**Figure 7:**
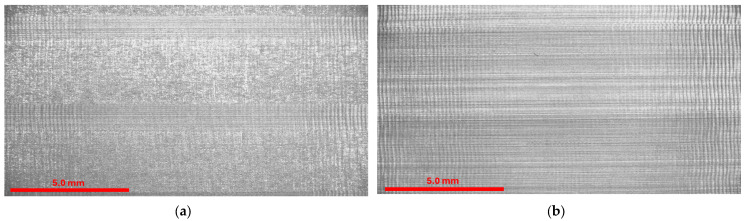
Manufactured surface: (**a**) R85; (**b**) R90.

The first step in evaluating the surfaces was evaluating them under a microscope. In Figure 7a,b, it is possible to see the surfaces obtained when machining with the lowest feed and with the smallest addition to the workpiece.

**Figure 8:**
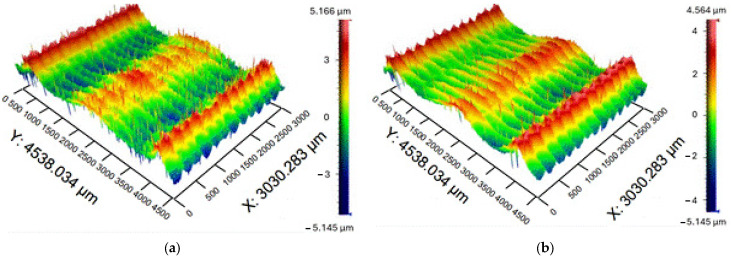
Scans of surfaces: (**a**) R85; (**b**) R90.

The area in the center of the machined surface was evaluated and measured to be 4.5 × 3 mm. The scans (Figure 8) showed that, indeed, two areas were obtained. This happened as a result of the large contact area of the tool when the tip of the tool had a lower cutting speed during the cutting process, and thus two different areas were created on the machined surface.

**Figure 9:**
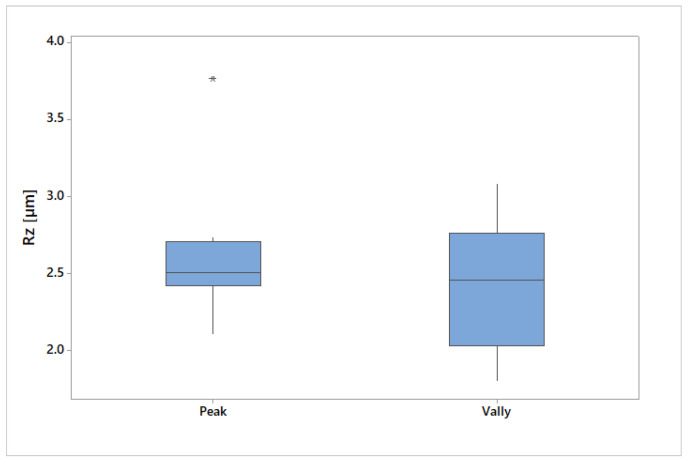
Comparison of roughness.

The slumped area created by the larger diameter of the tool was marked as Valley, and the raised area machined by the tool’s tip was marked as Peak. Figure 9 shows a boxplot showing the difference between the roughness sets measured in the Peak and Valley areas. It can be seen that the Peak region has less variance compared to the Valley region. This shows that there is a difference between the two areas and that their evaluations need to be done separately. In the evaluation, we can notice one outlier, which is displayed with an asterisk, and it was necessary to assess its correctness and re-evaluate it.

The parameters Ra and Rz were evaluated in terms of roughness. The individual parameters were entered into area graphs to simply describe the results. Plus, it is possible to determine which parameters to adjust to achieve the desired roughness.

**Figure 10:**
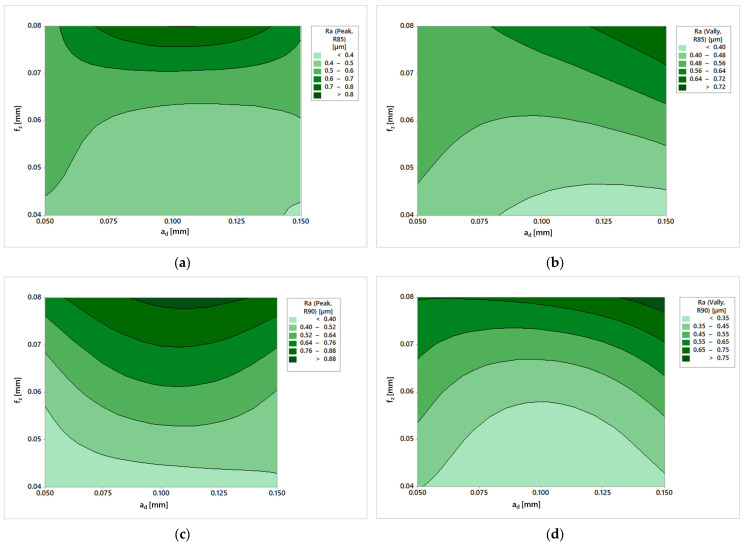
Ra results: (**a**) R85 in Peak area; (**b**) R85 in Valley area; (**c**) R90 in Valley area; (**d**) R90 in Valley area.

The first such graph is shown in Figure 10a. It can be seen from this figure that the effect of feed is more pronounced than the effect of depth of cut. The roughness varies slightly for surfaces machined with the same machining allowance. From this, it can be concluded that to achieve better roughness, it is more appropriate to adjust the feed, not the machining allowance.

In the Valley area (Figure 10b), the Ra parameter shows different trends than in the Peak area. Furthermore, it was confirmed that these are indeed heterogeneous surfaces. Here, at the mean value of the depth of cut (0.1 mm), there was a drop in the values for all the feeds used. It is therefore possible to claim that in this area, the depth of cut plays a more significant role, and it is necessary to find a compromise between the Valley and Peak areas so that both meet the specified parameters.

The same procedure was followed in the case of the second tool. The obtained results are shown in Figure 10c,d. The results of the R90 tool show greater regularity than those of the R85 tool in the Peak and Valley areas.

It is interesting to note that between Figure 10a,c, which show the Ra parameter in the Peak area, there is a noticeable difference in the tools used. While R85 shows the smallest roughness value only in a small area, in R90, this area is significantly larger. On the other hand, the other roughness values have a much narrower range of conditions at which they can be achieved. Another finding is that the dispersion of the achieved roughness is more significant in the case of the R90 tool, which also achieved higher values under the corresponding conditions. However, both tools can achieve Ra values of less than 0.4 µm under suitable conditions.

When comparing the Valley areas (Figure 10b,d), it is possible to observe similar results as those for the Peak areas, especially for the R90 tool and its dispersion of potential conditions for achieving the required roughness. Process optimization to achieve better Ra parameters will be more complicated for the R90 tool. The Peak and Valley areas show opposite trends; for example, a feed of 0.06 mm and an addition of 0.1 mm will lead to significantly different roughnesses. However, with the R85 tool, it is possible to find a setting where the Peak and Valley areas will only differ minimally.

However, both surfaces obtained are of high quality; a roughness Ra of 0.4 is usually achieved by grinding. Much higher values are generally achieved in milling, which requires a longer machining time. Thus, barrel cutters appear optimal for machining free surfaces because they shorten the production time and reduce the additional production costs associated with finishing operations.

From the evaluation, it was concluded that machining with a barrel tool resulted in the formation of a heterogeneous surface. This is more apparent in Figure 10, which was machined with tool R85. In the case of the R90 tool, the difference on the surface is not so noticeable.

**Figure 11:**
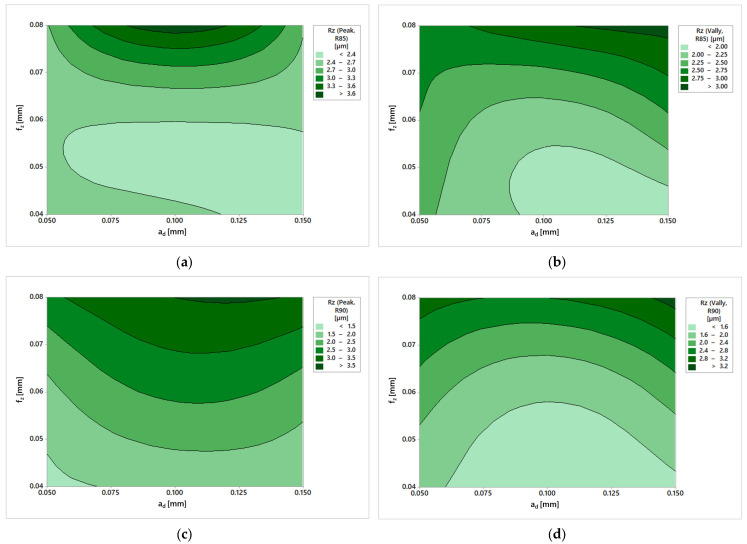
Rz results: (**a**) R85 in Peak area; (**b**) R85 in Valley area; (**c**) R90 in Valley area; (**d**) R90 in Valley area.

At first glance, the most striking difference in the Rz parameter of surfaces machined with the R85 tool (Figure 11a,b) is that the peak area again reaches higher values. However, this time, the difference is more significant. An interesting result is the relatively large areas of the two smallest Rz values in the Peak area. At the same time, the area where changes in the resulting roughness are radically different is from a feed value of 0.07. A possible recommendation would be to not exceed this feed value.

In the Peak and Valley areas, it is interesting that the smallest feed and feed do not necessarily lead to the lowest Rz parameter. This result emphasizes the importance of selecting appropriate cutting parameters during machining. Although the general rule is that a smaller feed and feed lead to better roughness, it has been shown here that there are limits to this effect.

A similar result was observed for the R90 tool. In the Peak area (Figure 11c), higher values and a more significant variation in results were recorded compared to the Valley area (Figure 11d). Due to the difference in cutting speed caused by the tool’s shape, more pronounced tool marks formed at the tool’s tip, leading to a significant increase in the Rz parameter.

When comparing the Rz parameter in the Peak area (Figure 11a,c), similar trends to those of the Ra parameter can be observed. The R90 tool demonstrated greater consistency in Rz distribution within these areas. In contrast, the R85 tool appears to have a higher tolerance for achieving low roughness values at lower feed rates. However, when the limits are exceeded, more drastic changes in results occur. Based on the curvature of the individual areas, it can be inferred that the influence of material allowance on machining is more pronounced for the R90 tool than for the R85 tool.

A comparison of the parameter Rz in the Valley area (Figure 11b,d) shows the same results in the Peak area, as was observed for the parameter Ra. The results of the R90 tool are more regularly and symmetrically distributed over all feed values per tooth. The R90 tool is therefore more suitable for use in areas of higher feed rates because even though the resulting surface roughness will be higher, the R90 tool offers a broader field to achieve the desired results. The R85 tool is suitable for lower feeds as its areas of lowest roughness are more significant than the R90 tool and it allows for larger machining allowances. The achieved values of the Rz parameter also indicate a high-quality surface. The usual Ra to Rz ratio for machined surfaces ranges from 1:4 to 1:7. In the case of surfaces machined with tangential tools, the ratio was at the lower limit of this range. Such a high-quality surface could already be sufficient, e.g., for mold cavities, as it would already meet the requirements for the product’s appearance.

As demonstrated in earlier works [10,16,17,18,19,20], the relationship between cutting conditions and both surface roughness and cutting forces in cylindrical milling of freeform surfaces is governed by a complex, multifactorial interaction involving cutting speed, feed rate, depth of cut, and tool geometry, including cylinder curvature and the effective contact path. The geometry of the cylindrical tool substantially influences the contact mechanics and the resulting surface topography, as the curvature radius and the distinction between inner (concave) and outer (convex) surfaces directly affect both the resulting roughness parameters and the magnitude of cutting forces.

For all investigated materials, increasing the feed and depth of cut generally leads to higher cutting forces and surface roughness, while higher cutting speeds frequently promote reductions in cutting forces and surface roughness, although material-specific behavior and coating characteristics may lead to deviations from this trend. The identification of optimal machining conditions therefore requires advanced experimental planning and dynamic stability assessments to define robust operating windows that balance minimized roughness with controlled cutting force levels in cylindrical milling operations. This is consistent with the results presented in this study.

## 4. Conclusions

This article describes the design and machining of freeform surfaces using two-barrel tools. The experiment was performed under specific cutting conditions: three feeds per tooth and three material allowance values. This approach allowed for the demonstration of their influence and the determination of the expected values for the monitored parameters.

Barrel cutters proved to be ideal for machining freeform surfaces. Their geometry enables a large contact area with the workpiece, significantly accelerating manufacturing. Additionally, finishing operations do not impose high cutting forces on the workpiece, which could otherwise lead to damage, especially to delicate components. The total cutting forces reached a maximum of 30 N, indicating that the vast majority of processes using these tools will not be negatively affected in this regard.

The surface roughness analysis revealed that machining with barrel tools results in a heterogeneous surface due to variations in cutting speeds along the tool’s contact length. As a result, the evaluation was carried out separately for both identified surface areas. The results showed that the area labeled as Valley exhibited higher quality, which can be attributed to the fact that it was machined with a larger tool diameter for which the experimental cutting conditions were optimized. Conversely, the Peak area, machined with the tool’s tip, showed higher roughness values and more significant variability in the achieved roughness results. Despite these variations, the overall surface quality remained high, suggesting that using barrel tools in practical applications can reduce production costs.

From a process quality control perspective, the machined surface’s heterogeneity necessitates precise measurement location specification to ensure that the assessed surface quality meets the required standards.

The findings concerning cutting forces and surface quality parameters are consistent with previously published research. Nonetheless, a more detailed assessment of the machined surface requires a new perspective on the identified surface heterogeneity.

It was also observed that, although the geometric differences between the tools may seem minor or negligible, they significantly impact both cutting forces and surface roughness. Cutting force variations of up to 30% were recorded. However, as mentioned earlier, the absolute magnitude of these forces should not pose any issues, such as potential workpiece damage. Differences in the measured roughness values were minor, and the final machined surface can still be considered exceptionally high quality.

## Figures and Tables

**Table 1:** Chemical composition of 1.1730.

Element	Value	Limit Values
C	0.45	0.40–0.50
Mn	0.7	0.60–0.80
Si	0.3	0.15–0.40
P	0.029	≤0.035
S	0.027	≤0.035

**Table 2:** Tool parameters.

	JH734100X2R2R85.0Z4 SIRA	207525 10/90	Unit
Cutting diameter	10	10	[mm]
Overall length	72	80	[mm]
Face cutting-edge count	4	4	[-]
Flute Helix Angle	20	30	[°]
Profile radius	85	90	[mm]
Depth of cut maximum in feed direction side	22.3	24.5	[mm]

**Table 3:** Measurement conditions.

Label	Parameter	Value			Unit
a_d_	Completion allowance	0.05	0.1	0.15	[mm]
f_z_	Feed per tooth	0.04	0.06	0.08	[mm/t]
v_c_	Cutting speed		200		[m/min]

**Table 4:** Comparison of cutting forces.

f_z_ [mm]	0.04			0.06			0.08		
a_d_ [mm]	0.05	0.1	0.15	0.05	0.1	0.15	0.05	0.1	0.15
Fc R85 [N]	6.19	11.6	17.73	8.35	15.93	23.43	10.4	19.3	29.07
Fc R90 [N]	8.26	14.74	21.28	10.58	18.76	26.98	12.58	22.76	32.46
Difference [%]	33.44	27.07	20.02	26.71	17.77	15.15	20.96	17.93	11.66

## Data Availability

The original contributions presented in this study are included in the article. Further inquiries can be directed to the corresponding author.
